# A new technique inducing mitral valve regurgitation as an experimental porcine model of volume-overload induced heart failure

**DOI:** 10.1038/s41598-026-43623-4

**Published:** 2026-03-14

**Authors:** Sven L. Van Laer, Bo Goovaerts, Steven Laga, Michiel R. L. Tubeeckx, Siel Van den Bogaert, Hein Heidbuchel, Vincent F. M. Segers, Marc J. Claeys

**Affiliations:** 1 Research Group of Cardiovascular Diseases (CARDIOVASC), University of Antwerp, Antwerp, Belgium; 2 Department of Cardiology, Antwerp University Hospital, Edegem, Belgium; 3 Laboratory of Physiopharmacology (FYSFAR), University of Antwerp, Antwerp, Belgium; 4 Department of Cardiac surgery, Antwerp University Hospital, Edegem, Belgium; 5 Research Group of Cardiovascular Diseases (CARDIOVASC), University of Antwerp, Drie Eikenstraat 655, Edegem, 2650 Belgium

**Keywords:** Cardiovascular Surgery, Heart Failure, Large Animal Model, Mitral Valve Insufficiency, Porcine Model, Translational Research, Cardiology, Diseases, Medical research

## Abstract

Heart failure (HF) is a common disease resulting in high morbidity, mortality, and healthcare costs. An important cause of HF is mitral valve regurgitation (MR), which induces left ventricular remodeling and volume overload. For HF research, a reproducible and reliable large animal model is crucial. Several MR models have been developed, but they often show high variability in MR severity and MR jet characteristics, as well as an underlying ischemic cardiomyopathy, which may increase the risk of complications during follow-up. We present a straightforward and uniform porcine model of primary MR-induced HF. A custom-made retractor was used to induce severe and uniform MR by chordal rupture. After four weeks, severe MR led to significant left ventricular remodeling compared to the sham group, with a significant increase in left ventricular end-diastolic (+34.5 ± 6.8 ml vs. +8.4 ± 5.3 ml, *p* = 0.023) and end-systolic volume (+29.9 ± 4.0 ml vs. +4.2 ± 2.9 ml, *p* = 0.001), a decrease in left ventricular ejection fraction (-11.9 ± 1.9% vs. -0.7 ± 0.9%, *p* < 0.001) and fractional shortening (-12.2 ± 1.3% vs. -2.2 ± 0.8%, *p* < 0.001), and a higher amount of left ventricular fibrosis (12.6 ± 1.0% vs. 6.4 ± 0.9%, *p* = 0.001). This porcine model allows a straightforward and reproducible induction of primary MR-induced HF, with a high success rate. It could be valuable in basic research to study the underlying pathophysiology and in translational research to develop novel diagnostics and therapeutics targeting volume-overload induced HF and/or primary MR.

**Supplementary Information:**

The online version contains supplementary material available at 10.1038/s41598-026-43623-4.

## Introduction

Heart failure (HF) is a prevalent disease which affects more than 64 million patients worldwide, leading to high morbidity, mortality, and healthcare costs. The prevalence is expected to further increase due to an ageing population^1–3^. Therefore, decreasing its medical and economic burden has become a major health care priority^2,3^. Mitral valve regurgitation (MR) is a common valvular heart disease with a global prevalence of over 2% in the general population and is known to cause clinical HF due to left ventricular remodeling and volume overload^4–8^.

A reproducible and reliable large animal model is crucial to further explore diagnostic and therapeutic advancements in the field of volume-overload induced HF and to translate these advancements from bench to bedside. Several ischemic models of secondary MR have been developed in large animals. These techniques induce ischemic cardiomyopathy, which in turn causes HF due to left ventricular dysfunction and remodeling. Ischemic induction of papillary muscle dysfunction, by occlusion or ligation of a marginal branch of the coronary arteries or by coronary ethanol infusions, has the potential of achieving good survival rates, depending on protocol optimization^9–12^. However, despite relatively low mortality rates, the degree of MR is more variable and often moderate, as has been shown previously^9,11^. Percutaneous techniques to induce primary MR via carotid or femoral approach have also been described^12–15^. However, the direction of the MR jet is difficult to standardize because selective transection of a chorda tendinea is challenging, mainly due to limited steerability and stability of the catheter-based device used in these techniques^12,13^.

The porcine heart is similar in physiology and pathophysiology to the human heart, and therefore a porcine model has a high translational potential^16–18^. The aim of the current study was to develop and validate a reproducible, reliable, and short-term large animal model of volume-overload induced HF without concurrent (ischemic) heart disease. To this end, we developed a straightforward technique to induce acute and severe primary MR using chordal transection under direct epicardial ultrasound imaging.

## Methods

### Ethical approval

All animal experiments were conducted in accordance with the European Convention for the Protection of Vertebrate Animals Used for Experimental and Other Scientific Purposes and were regulated by the institutional Ethics Committee of the University of Antwerp, Antwerp, Belgium (Case number: 2023-94). All responsible individuals involved in this experimental study underwent necessary education and are holders of an appropriate Federation of European Laboratory Animal Science Associations (FELASA) certificate. The authors complied with the ARRIVE guidelines.

### Animals and housing

Twenty-five (13 male and 12 female) healthy 16-weeks-old stress negative Piétrain pigs (*Sus scrofa domesticus*, RA-SE Genetics, Ooigem, Belgium) with an average initial weight of 43.6 ± 5.4 (36.5–56.0) kg were included in this study. The animals were acclimated for a minimum of 72 h and were housed in individual cages (with the ability to interact with each other) at 20–24 °C in a secluded building. There was a twelve-hour day-night cycle with additional daylight through a window. Cages were provided with synthetic coating over the full berth and stall mats at the lying areas, as well as cage enrichment and small amounts of hay to root in. The animals had free access to food and water, and they were fed a commercial standard pig diet (Carfil quality, Oud-Turnhout, Belgium). A total of 17 (9 male and 8 female) pigs underwent induction of severe MR, and 8 (4 male and 4 female) pigs underwent a sham procedure.

### Anesthesia, intubation and preparation

All animal experiments were performed under general anesthesia. The pigs were fasted for twelve hours with ad libitum access to water. They were sedated with an intramuscular injection of 0.5 mg/kg midazolam hydrochloride (Accord Healthcare, Stockley Park, UK) and 10 mg/kg ketamine (Ketalar^®^, Pfizer Inc., New York City, NY, USA), after which bodyweight was measured, and the animals were transported to the operating room using a hammock on wheels. ECG monitoring, pulse oximetry, and thermometry were connected, and an over-the-needle catheter (18 G) was inserted into a marginal ear vein.

For induction, an intravenous (IV) bolus of 1–4 mg/kg propofol (Propofol^®^ MCT, Fresenius Kabi, Bad Homburg, Germany) was administered. The pig was placed in prone position, after which an endotracheal tube (ETT) with an internal diameter of 7 mm was introduced using a standard laryngoscope and a stylet while the pig’s mouth was held open by an assistant. The pig was then ventilated (tidal volume of 10 ml/kg, peak inspiratory pressure of 11–15 cmH_2_0, positive end-expiratory pressure of 2–5 cmH_2_0, respiratory rate of 12–16 breaths per minute to maintain end-tidal CO_2_ between 35 and 45 mmHg, FiO_2_ of 50% (which was reduced when oxygen saturation reached 100%)), and gas-maintained anesthesia was provided with sevoflurane 2.5% (Sevorane^®^, AbbVie Inc., North Chicago, IL, USA). For analgesia, a continuous rate infusion of 2–5 µg/kg/h alfentanil (Rapifen^®^, Janssen-Cilag, Beerse, Belgium) was used. Fluid management was performed with 3–5 ml/kg/h Plasma-Lyte 148 (Baxter, Deerfield, IL, USA). Antibiotic prophylaxis was given as 1 g IV cefazoline (Viatris Inc., Canonsburg, PA, USA) and for every additional two hours of surgery, an extra 500 mg of cefazoline was administered. The thorax of the pig was shaven, and the vital parameters and depth of anesthesia were continuously monitored throughout the procedure by an experienced veterinary anesthesiologist. The pig was positioned in a supine position, after which both groins were disinfected with chlorhexidine. An arterial catheter (5 Fr) for continuous arterial blood pressure monitoring and a central venous catheter (8 Fr) for blood sample collection/administration of drugs were placed in the femoral artery and vein using the Seldinger technique with ultrasound guidance. The sheaths were fixated with a polyester suture (Mersilene™ 0, Ethicon, Johnson & Johnson Medical, New Brunswick, NJ, USA).

### Surgical method

#### Induction of severe MR

The skin was rigorously disinfected using iodine 2% and sterile drapes were applied around the surgical field. A median incision was made from the sternal manubrium to 3 cm below the xiphoid process. The visceral sternal surface was disconnected from the connective tissue by blunt dissection. Sternotomy was performed with a sternal saw, a sternal retractor was inserted, and the pericardium was opened, which was kept in place with suspension sutures.

**Fig. 1:**
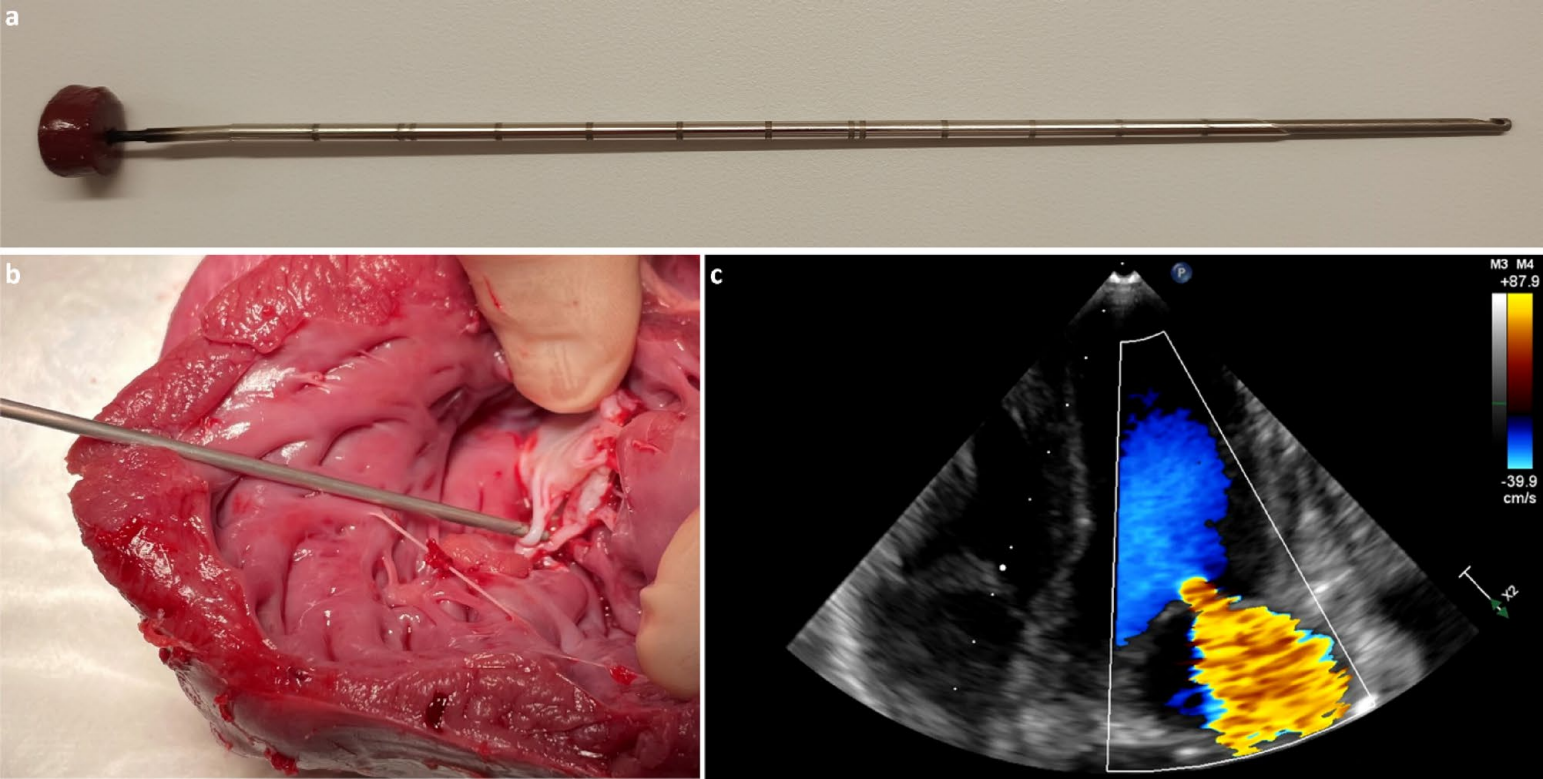
Custom-made retractor to induce severe mitral valve regurgitation. (**a**) The custom-made retractor, consisting of a 14 G sharp biopsy needle, (taken from a Tru-Cut® device, (Merit Medical Systems, South Jordan, UT, USA)) to puncture the left ventricular apex, and an atraumatic hook, (taken from an Endo Close™ trocar site closure device, (Medtronic, Fridley, MN, USA)) which is pushed out to grab a chorda tendinea of the mitral valve, which is then retracted in the needle to be cut. (**b**) The custom-made retractor inserted through the left ventricular apex in an isolated heart grabbing one of the chordae tendineae of the mitral valve. (**c**) Epicardial ultrasound four chamber view showing a severe mitral valve regurgitation after cutting one of the chordae tendineae of the mitral valve.

A custom-made retractor, consisting of a 14 G biopsy needle (taken from a Tru-Cut^®^ device (Merit Medical Systems, South Jordan, UT, USA)) over an atraumatic hook (taken from an Endo Close™ trocar site closure device (Medtronic, Fridley, MN, USA)) that can be advanced or retracted (Fig. 1a), was inserted through the left ventricular apex. A purse string suture with polypropylene (Prolene™ 6 − 0, Ethicon, Johnson & Johnson Medical, New Brunswick, NJ, USA) was left in place. Under direct vision of the left ventricle, and guided by epicardial ultrasound, one of the chordae tendineae of the anterior mitral valve leaflet was grasped and retracted to assess for an eccentric MR jet toward the posterior wall. If the direction and severity of the jet were satisfactory, the chorda was transected to induce significant MR (Fig. 1b). If the direction of the MR jet was not appropriate or if the jet was too severe, the chorda was released, and another chorda was targeted for transection. If the MR was not considered severe based on the echocardiographic criteria discussed below, the described procedure was repeated a second time. After successful induction of severe MR (Fig. 1c), the retractor was removed while closing the wound by tightening the purse string suture.

A drain was left in the mediastinum and was tunneled to the skin surface. The pericardium was closed with polypropylene sutures (Prolene™ 6 − 0, Ethicon, Johnson & Johnson Medical, New Brunswick, NJ, USA). The sternum was closed with stainless steel wire (Stainless steel 5, Ethicon, Johnson & Johnson Medical, New Brunswick, NJ, USA) using a cerclage technique. The subcutis was closed in two layers with polyglactin 910 sutures (Vicryl™ 1, Ethicon, Johnson & Johnson Medical, New Brunswick, NJ, USA). The skin was closed with a continuous intradermal suture using poliglecaprone (Monocryl™ 3 − 0, Ethicon, Johnson & Johnson Medical, New Brunswick, NJ, USA). Finally, a sternal block was initiated by administering 5 ml of bupivacaine 0.5 mg/ml + adrenaline 1/200.000 (Marcaine^®^, Aspen Pharmacare, Durban, South Africa) intradermally.

#### Sham procedure

The exact same protocol was performed (including the insertion of the retractor through the left ventricular apex), without the cutting of the chordae tendineae of the mitral valve.

### Echocardiographic assessment

The left atrial and left ventricular volumes, as well as left ventricular function, were quantified using epicardial echocardiography (Philips EPIQ 7c diagnostic ultrasound system, Philips Ultrasound Inc., Bothell, WA, USA). In addition, MR severity was assessed intraoperatively using the validated multi-integrative method according to the American Society of Echocardiography guidelines, including assessment of the vena contracta, as well as regurgitant volume (RV), regurgitant fraction (RF), and effective regurgitant orifice area (EROA) according to the proximal isovelocity surface area (PISA) method^19^. To complement the MR quantification, we also assessed the presence of a wall-impinging/swirling MR jet reaching the posterior wall of the left atrium (Coandă effect), indicating severe eccentric MR^20^.

### Postoperative course

A 50 µg/h fentanyl patch (Matrifen^®^, Takeda Pharmaceutical Company Limited, Tokyo, Japan) was applied for three days and 0.01 mg/kg buprenorphine (Vetergesic^®^, Ceva Animal Health, Libourne, France) was administered subcutaneously. The ETT was removed, as well as the catheters and mediastinal drain. The pig was continuously monitored within the first hours after awakening and if necessary, additional analgesia was administered. Antibiotic prophylaxis was given as 1 g IV cefazoline 8 and 16 h after surgery, and subsequently 25/5 mg/kg/day trimethoprim/sulfamethoxazole (Eusaprim^®^ suspension, Aspen Pharmacare, Durban, South Africa), as well as 10 mg/kg/day doxycycline (EG, Brussels, Belgium), were given per os for five days. To prevent and/or control HF symptoms, 4 mg/kg/day furosemide (Lasix^®^, Sanofi, Paris, France) was given per os until sacrifice after four weeks. Daily clinical examination was performed during follow-up.

### Euthanasia, tissue harvesting and histological examination

After four weeks, sternotomy was repeated and epicardial ultrasound was performed according to the protocol described above. After completion of the final experiment, the animals were euthanized using an overdose of 50 mg/kg IV pentobarbital sodium 300 mg/ml (Release^®^, Calier, Barcelona, Spain). Next, the heart was excised and transmural biopsies were taken from the mid-anterolateral wall of the left ventricle to quantify interstitial fibrosis using Masson’s trichrome staining. The stained sections were examined under a light microscope (Olympus BX43F upright microscope, Olympus, Tokyo, Japan). Five randomly selected digital images were acquired per section. Quantification of ventricular interstitial fibrosis was performed in a blinded manner using ImageJ image analysis software. For each image, the number of blue pixels was calculated and expressed as a percentage of the total number of non-white pixels.

### Statistical analysis

The sample size was calculated based on an estimated 15 ml difference in left ventricular end-diastolic volume (LVEDV) at four weeks in the MR group compared to the sham group, which serves as an indicator of volume overload. This difference corresponds to a 10–15% increase in LVEDV compared to baseline measurements, which is considered a clinically relevant marker of left ventricular remodeling and an independent risk factor for cardiovascular outcomes in systolic HF^21,22^. With an estimated standard deviation of 10 ml, a type 1 error of 0.05, a type 2 error of 0.20, and a 2:1 randomization ratio to the MR group vs. the sham group, a sample size of 18 pigs (12 MR + 6 sham) is required to prove the difference in LVEDV. All continuous variables were tested for normality with the Kolmogorov-Smirnov test and by visual assessment of the Quantile-Quantile plots and histograms. Continuous variables are presented as mean ± standard error of the mean or median (interquartile range) in case of nonparametric distribution. Between-group comparisons, including the amount of left ventricular interstitial fibrosis, were performed using an independent samples T-test (or Mann-Whitney U test for nonparametric testing). A repeated measures ANOVA (or Friedman’s test) was performed to compare baseline echocardiographic measurements with those after four weeks of follow-up between both groups, with missing data being automatically excluded from the analyses. To test for sex effect on group differences, a two-way ANOVA with group, sex, and group by sex interaction, was performed. A two-tailed p-value < 0.05 was considered statistically significant. Statistical analyses were performed using MedCalc version 20.0 for Windows (MedCalc Software, Ostend, Belgium).

## Results

We observed peri-operative mortality in three pigs in the intervention group due to intraoperative cardiogenic shock; sudden cardiac death one hour postoperatively; and cardiogenic shock after two and a half weeks of follow-up, and one pig in the control group due to cardiorespiratory collapse on induction. Autopsy of this control pig showed a laryngeal dislocation, probably caused by accidental neck trauma during routine caretaking. Due to technical issues with our ultrasound system, echocardiographic imaging during follow-up was unreliable in two pigs of the intervention group and one pig of the sham group. Therefore, these pigs were excluded from the echocardiographic analyses but were included in the anatomopathological analyses.

### Echocardiographic evaluation of MR severity and left atrial volume

**Fig. 2:**
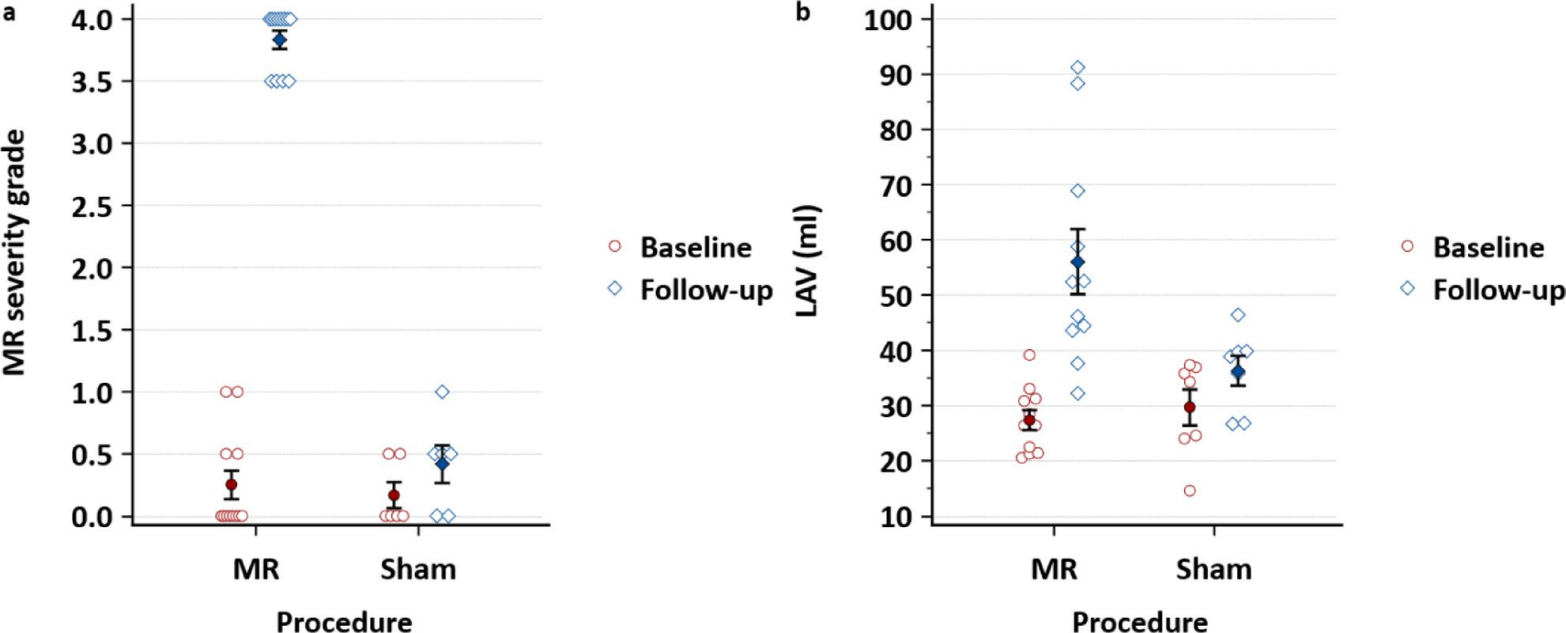
Evolution of MR severity and left atrial volume after four weeks. (**a**) The mitral valve regurgitation (MR) severity grade ± standard error of the mean (SEM) of the MR group (*n* = 12) vs. the sham group (*n* = 6) at baseline and four weeks after surgery (*p* < 0.001). (**b**) The mean left atrial volume (LAV) ± SEM of the MR group vs. the sham group at baseline compared to the mean LAV after four weeks (*p* = 0.011). All statistical analyses were performed using repeated measures ANOVA.

Echocardiographic evaluation at four weeks showed MR severity grade 3.5 to 4/4 in all pigs of the intervention group (Fig. 2a). The measurements on which this quantification is based are shown in Table 1. These measurements, combined with the presence of a Coandă effect in all pigs of the intervention group, indicate severe eccentric MR.

The pigs in the MR group developed significant left atrial myopathy. Severe MR led to left atrial dilation, with a significantly greater increase in mean left atrial volume (LAV) at four weeks compared to the sham group (+28.1 ± 5.4 ml vs. +5.4 ± 2.0 ml, *p* = 0.011; Fig. 2b). No significant sex differences were observed.

**Table 1:** Semi-quantitative and quantitative assessment of MR severity in the intervention group after four weeks.

Parameter	Value
Vena contracta (mm)	6.94 ± 0.22
PISA radius (mm)	6.61 ± 0.47
EROA (cm²)	0.22 ± 0.03
RV (ml)	23.80 ± 3.32
RF (%)	40.85 ± 2.99

EROA: effective regurgitant orifice area, MR: mitral valve regurgitation, PISA: proximal isovelocity surface area, RF: regurgitant fraction, RV: regurgitant volume.

All continuous data are presented as mean ± standard error of the mean.

### Echocardiographic evaluation of left ventricular volumes, ejection fraction, and fractional shortening

**Fig. 3:**
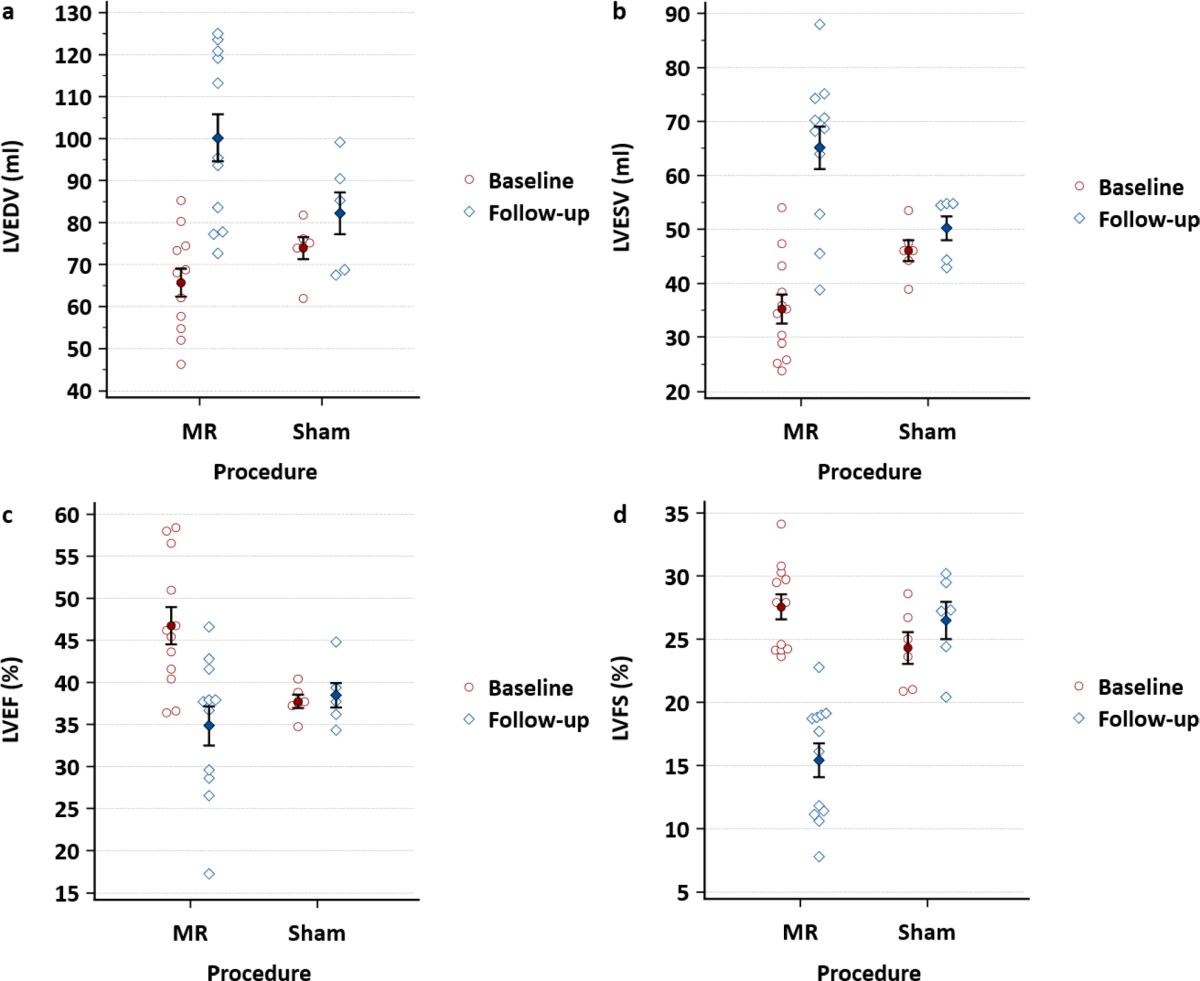
Evolution of left ventricular volumes, ejection fraction, and fractional shortening after four weeks. (**a**) The mean left ventricular end-diastolic volume (LVEDV) ± standard error of the mean (SEM) of the MR group (*n* = 12) vs. the sham group (*n* = 6) at baseline compared to the mean LVEDV after four weeks (*p* = 0.023). (**b**) The mean left ventricular end-systolic volume (LVESV) ± SEM of the MR group vs. the sham group at baseline compared to the mean LVESV after four weeks (*p* = 0.001). (**c**) The mean left ventricular ejection fraction (LVEF) ± SEM of the MR group vs. the sham group at baseline compared to the mean LVEF after four weeks (*p* < 0.001). (**d**) The mean left ventricular fractional shortening (LVFS) ± SEM of the MR group vs. the sham group at baseline compared to the mean LVFS after four weeks (*p* < 0.001). All statistical analyses were performed using repeated measures ANOVA.

There was evidence of left ventricular remodeling and dysfunction in the MR group after four weeks, which showed a significant increase in mean LVEDV compared to the sham group (+34.5 ± 6.8 ml vs. +8.4 ± 5.3 ml, *p* = 0.023; Fig. 3a), as well as an increase in mean left ventricular end-systolic volume (LVESV, +29.9 ± 4.0 ml vs. +4.2 ± 2.9 ml, *p* = 0.001; Fig. 3b), and a decrease in mean left ventricular ejection fraction (LVEF, −11.9 ± 1.9% vs. −0.7 ± 0.9%, *p* < 0.001; Fig. 3c), as well as a decrease in mean left ventricular fractional shortening (LVFS, −12.2 ± 1.3% vs. −2.2 ± 0.8%, *p* < 0.001; Fig. 3d). On an individual basis, we observed a significant increase in LVEDV and LVESV, and a decrease in LVEF and LVFS in all pigs of the MR group, while this was not the case in the sham group (see Supplementary Fig. S1−4 online). No differences were observed between female and male pigs.

### Histological examination of left ventricular interstitial fibrosis

**Fig. 4:**
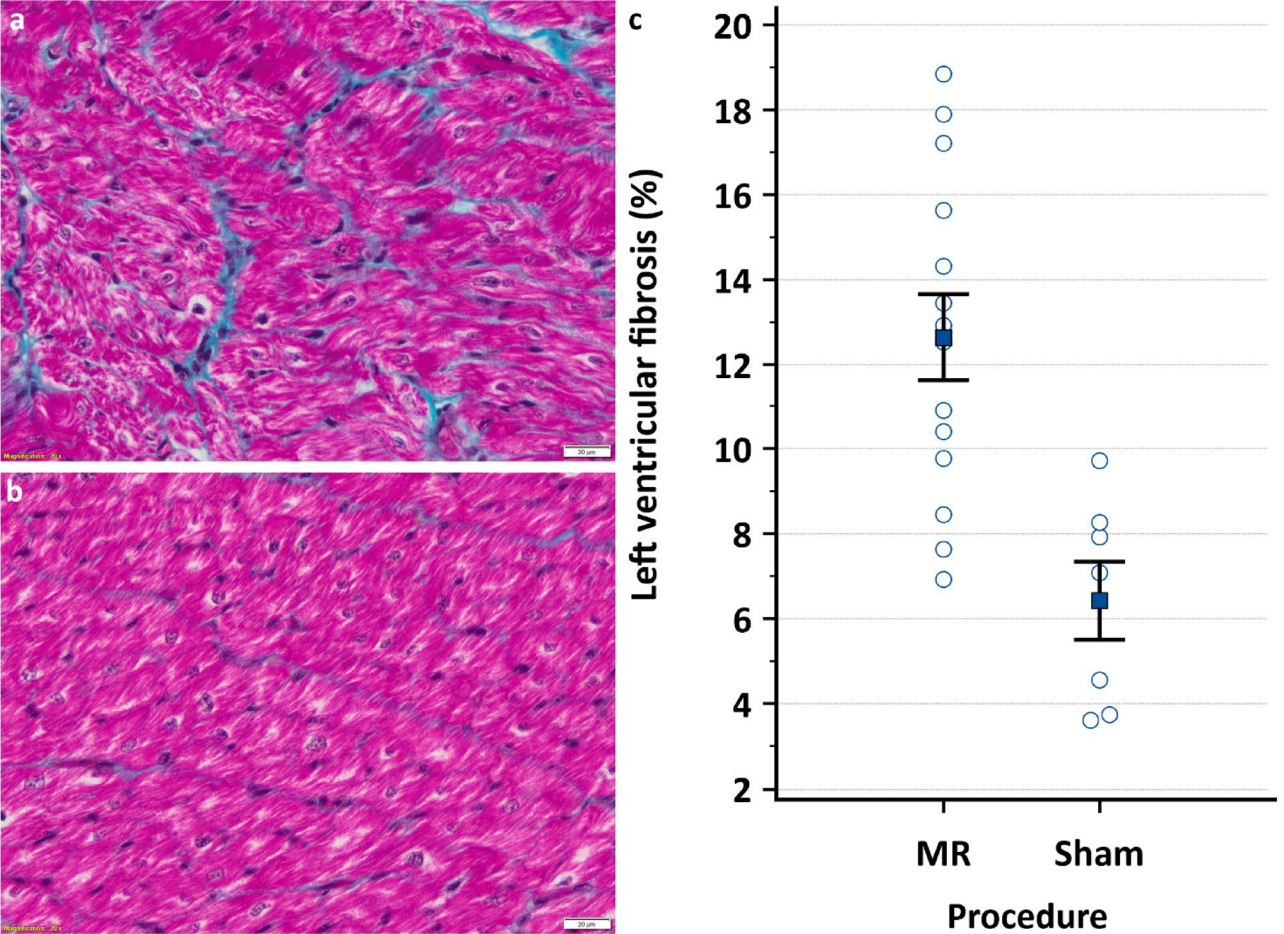
Left ventricular interstitial fibrosis after four weeks. (**a**) Masson’s trichrome staining of left ventricular epicardial and myocardial tissue of a pig in the severe mitral valve regurgitation (MR) group. Blue color represents fibrotic tissue, magnification: 40x; scale bars: 20 μm. (**b**) Masson’s trichrome staining of left ventricular epicardial and myocardial tissue of a pig in the sham group. Blue color represents fibrotic tissue, magnification: 40x; scale bars: 20 μm. (**c**) Blinded quantification of the mean percentage of left ventricular fibrotic tissue relative to the total tissue area ± standard error of the mean using ImageJ software in the MR group (*n* = 14) vs. the sham group (*n* = 7; *p* = 0.001; independent samples T-test).

The pigs with severe MR showed significantly more left ventricular interstitial fibrosis after four weeks compared to the sham group (12.6 ± 1.0% vs. 6.4 ± 0.9%, *p* = 0.001; Fig. 4). There was no significant sex difference.

## Discussion

Small animals, such as rodents, are often used for basic research because of their manageability and low cost. However, their cardiac anatomy, physiology, and pathophysiology differ significantly from that of humans^17,23^. A reliable and straightforward large animal model is therefore essential to study MR and HF and to develop novel therapeutic agents for both pathologies. Stress-negative Piétrain pigs are the common commercial breeding pigs in our region of Europe. They are easy to manage, and the porcine heart is structurally and physiologically similar to the human heart. Furthermore, the use of Piétrain pigs is less cost-prohibitive compared to the use of minipigs^17,18^.

We were able to validate our MR model as a representative model of volume-overload induced HF. After four weeks, all individual pigs in the MR group developed significant left ventricular remodeling compared to the sham group, with a significant increase in LVEDV and LVESV, a decrease in LVEF and LVFS, and a higher degree of left ventricular interstitial fibrosis. Furthermore, there was evidence of significant left atrial remodeling in the MR group compared to the sham group, with a significant increase in LAV. The pathophysiology induced in this model also closely resembles the pathophysiology observed in human patients, as left ventricular dilation and fibrosis precede the occurrence of clinical HF, further increasing the translational value of our model^2–8^. We included both female and male pigs in our study to correct for potential sex differences in cardiac remodeling, but found no sex differences in cardiac dimensions and left ventricular function, nor in the amount of left ventricular interstitial fibrosis.

The four-week follow-up period in the present model was chosen for practical reasons, including the difficulty of clinically monitoring the animals over an extended period given the pronounced severity of MR and HF, as well as cost-effectiveness. Furthermore, we were able to achieve a clinically relevant stage of volume-overload induced HF in all pigs within the relatively short timeframe of four weeks. This was defined as a 15% increase in LVEDV after four weeks compared to baseline measurements, which is representative of a phenotype of volume-overload induced HF^21,22^. However, we observed some heterogeneity in HF phenotype in the MR group, as nine of the twelve pigs achieved this increase in LVEDV after four weeks. The other three pigs all showed a decrease in systolic left ventricular function with a more than 10% decrease in LVEF, which is also a parameter for a clinically relevant phenotype of systolic HF^24,25^. Furthermore, all pigs in the MR group required a significant dose of loop diuretics during follow-up, unlike the pigs in the sham group. Taking all these factors into account, all pigs in the MR group reached the endpoint of a clinically relevant HF phenotype. A possible explanation for the variation in HF phenotype could be natural heterogeneity between pigs regarding remodeling/decompensation, despite similar MR severity. Moreover, differences in the loading conditions during echocardiographic measurements could have potentially influenced left ventricular echocardiographic measurements. Finally, it is expected that with increasing follow-up time, the severity of HF will further increase and the heterogeneity in left ventricular remodeling will further decrease.

Other experimental models of volume-overload induced HF include aortic valve regurgitation and aortocaval fistulas^26^. A few animal models of aortic valve regurgitation have been described, in which isolated, severe left ventricular volume overload occurs due to diastolic regurgitation of blood from the aorta into the left ventricle, after which this blood is pumped back into the aortic cavity against high pressure during systole^26,27^. Additionally, aortocaval fistula models have been described, which are primarily used in rodents, in which hyperdynamic circulation with biventricular volume overload occurs due to factors such as neurohormonal overstimulation^26,28^. In contrast, MR models cause combined left atrial and left ventricular volume overload due to systolic regurgitation of blood from the left ventricle into the left atrium, which is then pumped back into the left ventricle during diastole^12,13,26^.

Despite etiological differences, primary and secondary (ischemic) MR models induce similar progressive left ventricular remodeling and HF due to volume overload. However, in addition to volume overload, ischemic MR models also induce regional left ventricular dyskinesia due to ischemic cardiomyopathy, often resulting in more pronounced left ventricular systolic dysfunction compared to primary MR models^9,11,12^. In contrast, our primary MR design allows for a model of isolated volume-overload induced HF, so that the evaluation of left ventricular function and remodeling is not affected by concomitant (ischemic) heart disease, as is the case with secondary MR models. Furthermore, ischemic MR models are often characterized by more extensive left atrial remodeling due to concomitant left atrial ischemia^12^. Finally, the MR jet in secondary MR models is typically more centrally located compared to the eccentric jet resulting from chordal rupture, which is inherent in primary MR models^10,12^.

The current model of HF induced by primary MR due to chordal rupture is relatively straightforward compared to other secondary MR models described in the literature, such as ischemic MR models based on papillary muscle dysfunction. Due to the potential infarction of a larger part of the heart than intended, these techniques may lead to higher variability in MR severity/MR jet direction and additional procedure-induced left ventricular damage, which may complicate animal follow-up^9–12,16^. Our model shows high uniformity (with an MR severity of 3.5 to 4/4 in all pigs of the intervention group) and low mortality, provided a good follow-up of the animals. We therefore emphasize the importance of diuretic therapy to promote the transition of acute to chronic MR and prevent acute pulmonary edema, as well as adequate antibiotic prophylaxis in the postoperative period, given the animals’ susceptibility to develop respiratory infections.

Another open-chest model of primary MR was described by Li et al.^29^. They developed a method for inducing MR in a Guizhou minipig model by cutting the chordae tendineae of the posterior mitral valve leaflet with a self-made retractor^,^ which was inserted through the left atrial appendage. However, this technique does not allow the chordae to be cut under direct vision, which can compromise the degree of uniformity. Also, the retractor faces the atrial surface of the mitral valve, while the chordae are located on the ventricular surface, making the technique more difficult. This study group found no significant difference in left ventricular volumes and fibrosis in the MR group compared to the control group. This remarkable difference with our study protocol may be explained by the fact that the MR in the minipigs may have been less severe and less uniform. On the other hand, it could be hypothesized that minipigs, due to their smaller size and lower weight, are less likely to develop cardiac fibrosis and subsequent left ventricular remodeling compared to large Piétrain pigs.

Finally, a few large animal closed-chest models exist in which primary MR is induced by transecting the chordae tendineae of the mitral valve using a percutaneously introduced catheter-based device^12–16^. Our model has some limitations in terms of scalability due to its invasiveness compared to these percutaneous models. However, the quality of echocardiographic images in these models is expected to be suboptimal due to the retrosternal position of the left ventricular apex in pigs and the subsequent limited availability of ultrasound windows, which complicates the assessment of the effect of chorda transection and prevents the follow-up of extensive echocardiographic measurements^30^. Furthermore, the combination of limited catheter stability and steerability in these models can lead to significant variability in the direction of the MR jet, as it is challenging to target a specific chorda of one of the mitral valve leaflets. Sakata et al.^12^ report observing significant variation in the direction of the regurgitant jet in their closed-chest MR model. On the other hand, Watanabe et al.^13^ report a 30-days survival rate of only 47.6% in their catheter-based model after inducing severe MR. In contrast, our technique allows for epicardial ultrasound imaging, which provides excellent image quality. In addition, by puncturing directly through the left ventricular apex under direct vision, we are able to accurately steer our retractor and precisely determine which chorda we want to grasp. We can then make an excellent assessment of the expected effect of the chorda transection by first retracting it and assessing the resulting regurgitant jet live with epicardial echocardiography. Our technique provides greater uniformity in both the direction and characteristics of the MR jet compared to closed-chest models of primary MR.

The results of this study should be considered in light of the following limitations. First, the MR jets in our study were highly eccentric, given the primary etiology of MR due to chordal rupture. Quantifying the severity of eccentric MR often leads to an underestimation of MR severity, with vena contracta being the more reliable measure. Therefore, the Coandă effect, which reflects severe eccentric MR, was added to the assessment of MR severity and was present in all pigs of the intervention group. Second, although intraoperative epicardial ultrasound provides excellent image quality, an inherent limitation of this technique is the occurrence of left ventricular foreshortening due to poor visualization of the left ventricular apex. This can lead to an underestimation of the absolute values ​​of left ventricular dimensions and LVEF, resulting in some variability. To compensate for this, we added LVFS as a parameter of left ventricular remodeling, as LVFS is less susceptible to left ventricular foreshortening. Furthermore, we focused on changes in left ventricular dimensions and function after four weeks, rather than on absolute values, clearly demonstrating that pigs in the MR group developed a volume-overload induced HF phenotype, while this was not the case in the sham group. Moreover, differences in preload and/or afterload at the time of measurements could have potentially influenced left ventricular dimensions and LVEF. Although we attempted to standardize the depth of anesthesia, blood pressure, and heart rate as much as possible during echocardiographic assessment by continuously monitoring these parameters, we cannot completely rule out variability. Third, given the relatively limited number of subjects in the sex subgroups, it was not possible to make a definitive statement about the presence or absence of sex differences. However, to compensate for potential sex differences, equal numbers of male and female pigs were included in both the MR and sham groups, and no sex differences were observed. Finally, the generalizability of our results to common commercial pig breeds in other regions (e.g., Yorkshire, Large White, Landrace) is uncertain, as our study exclusively used stress-negative Piétrain pigs. However, we expect our results to be valid for other large pig breeds, given the surgical nature of our model.

## Conclusion

We have developed a novel and reproducible experimental porcine model of severe primary MR with high success rate, which serves as a reliable and valid model of volume-overload induced HF, as shown by significant left ventricular remodeling already occurring after four weeks. Therefore, our model is particularly valuable for extensive diagnostic and therapeutic in vivo experiments in the field of volume-overload induced HF and/or primary MR.

## Supplementary Information

Below is the link to the electronic supplementary material.

Supplementary Material 1

## Data Availability

The datasets generated and/or analyzed during the current study are available from the corresponding author on reasonable request.
