# Supplementary material for: A new technique inducing mitral valve regurgitation as an experimental porcine model of volume-overload induced heart failure
Source: Sci Rep. 2026 Mar 14;16:13500. doi: 10.1038/s41598-026-43623-4 (PMC13111651; doi:10.1038/s41598-026-43623-4)
Supplement: Supplementary file 1 — Supplementary Material 1 [file 41598_2026_43623_MOESM1_ESM.docx]

**Supplementary Information file 1**

**Supplementary Figure S1**


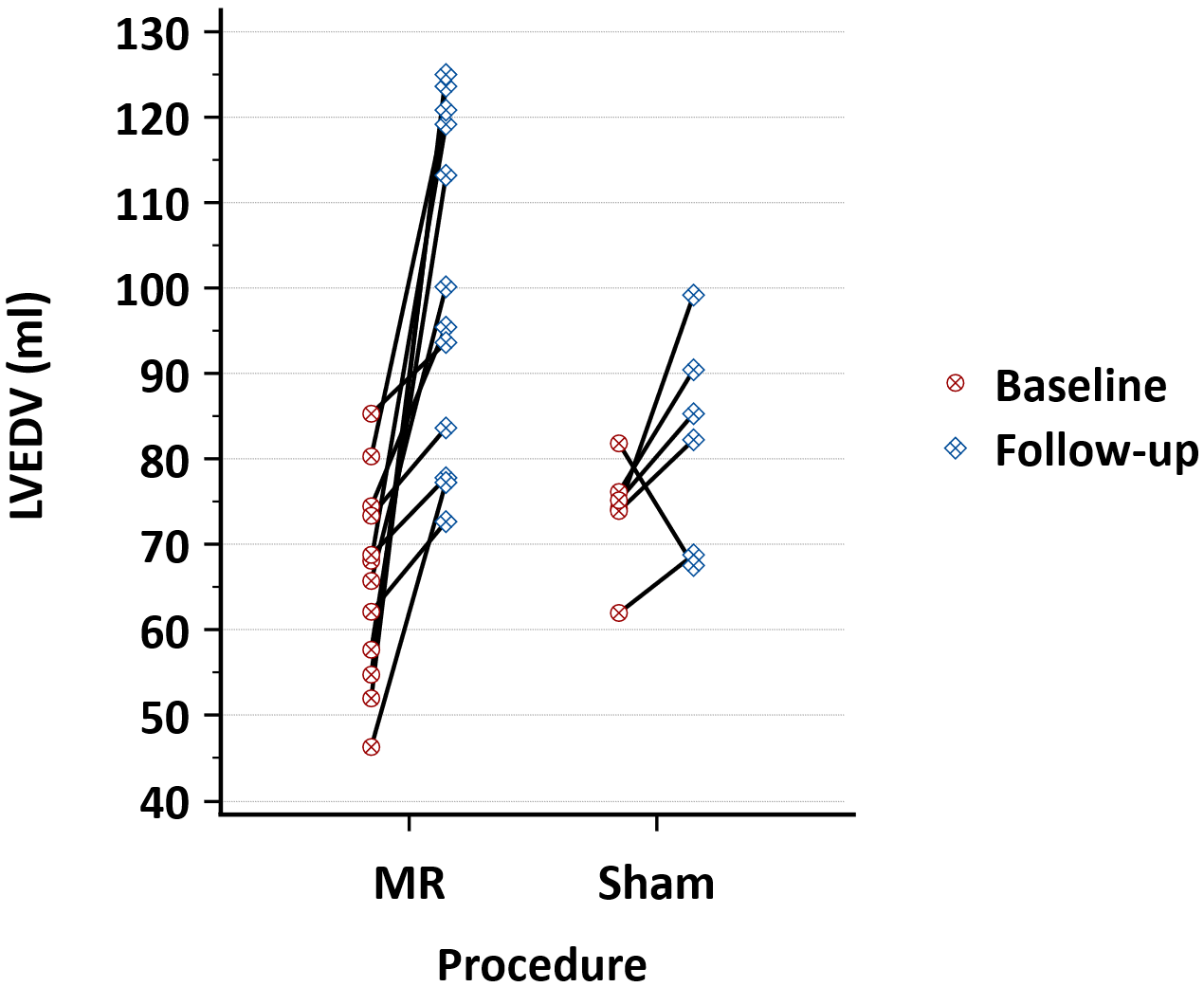


Individual evolution of left ventricular end-diastolic volume (LVEDV) over the four-week follow-up period for the MR group (n = 12) vs. the sham group (n = 6) (p = 0.023; repeated measures ANOVA).

**Supplementary Figure S2**


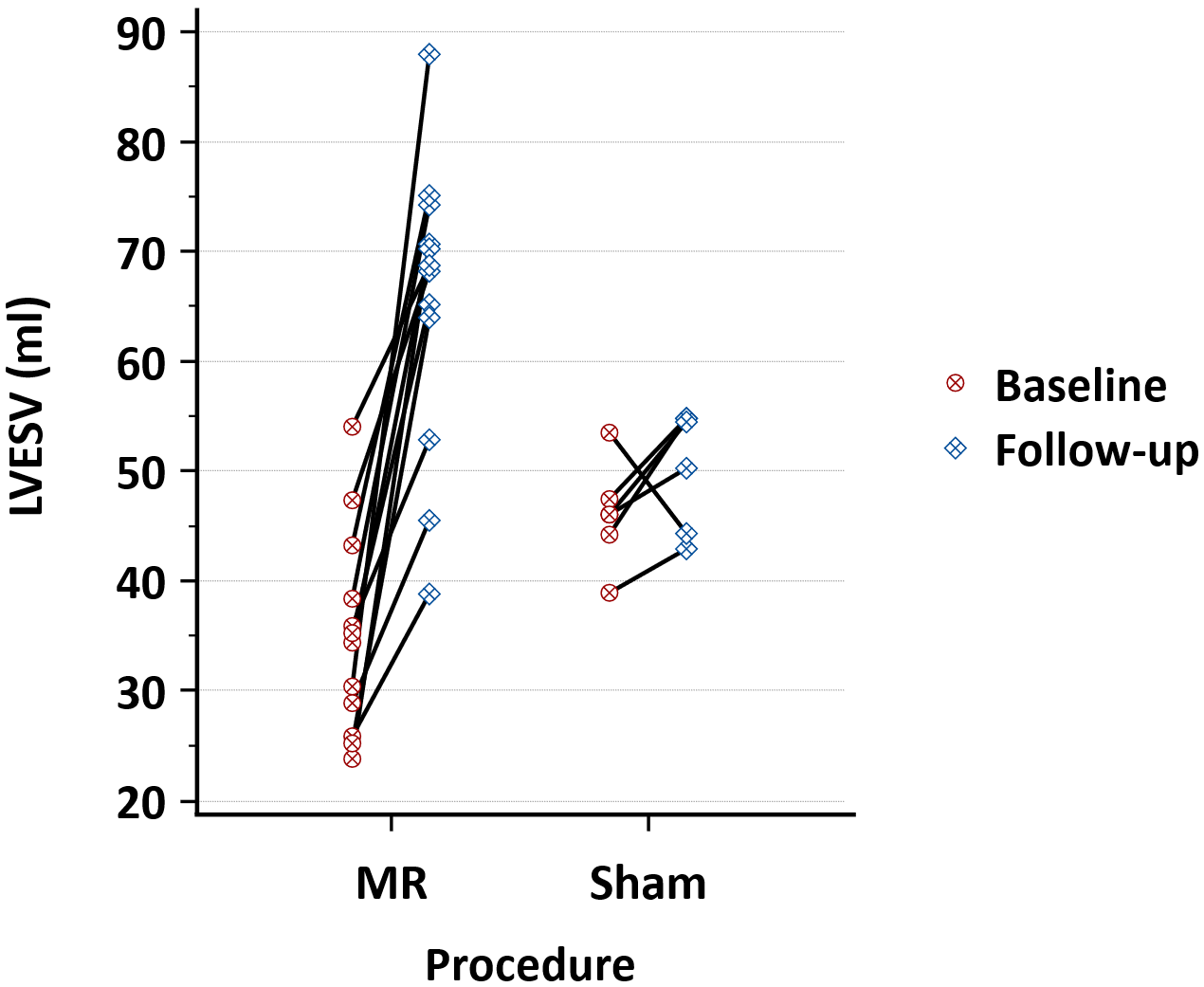


Individual evolution of left ventricular end-systolic volume (LVESV) over the four-week follow-up period for the MR group (n = 12) vs. the sham group (n = 6) (p = 0.001; repeated measures ANOVA).

**Supplementary Figure S3**


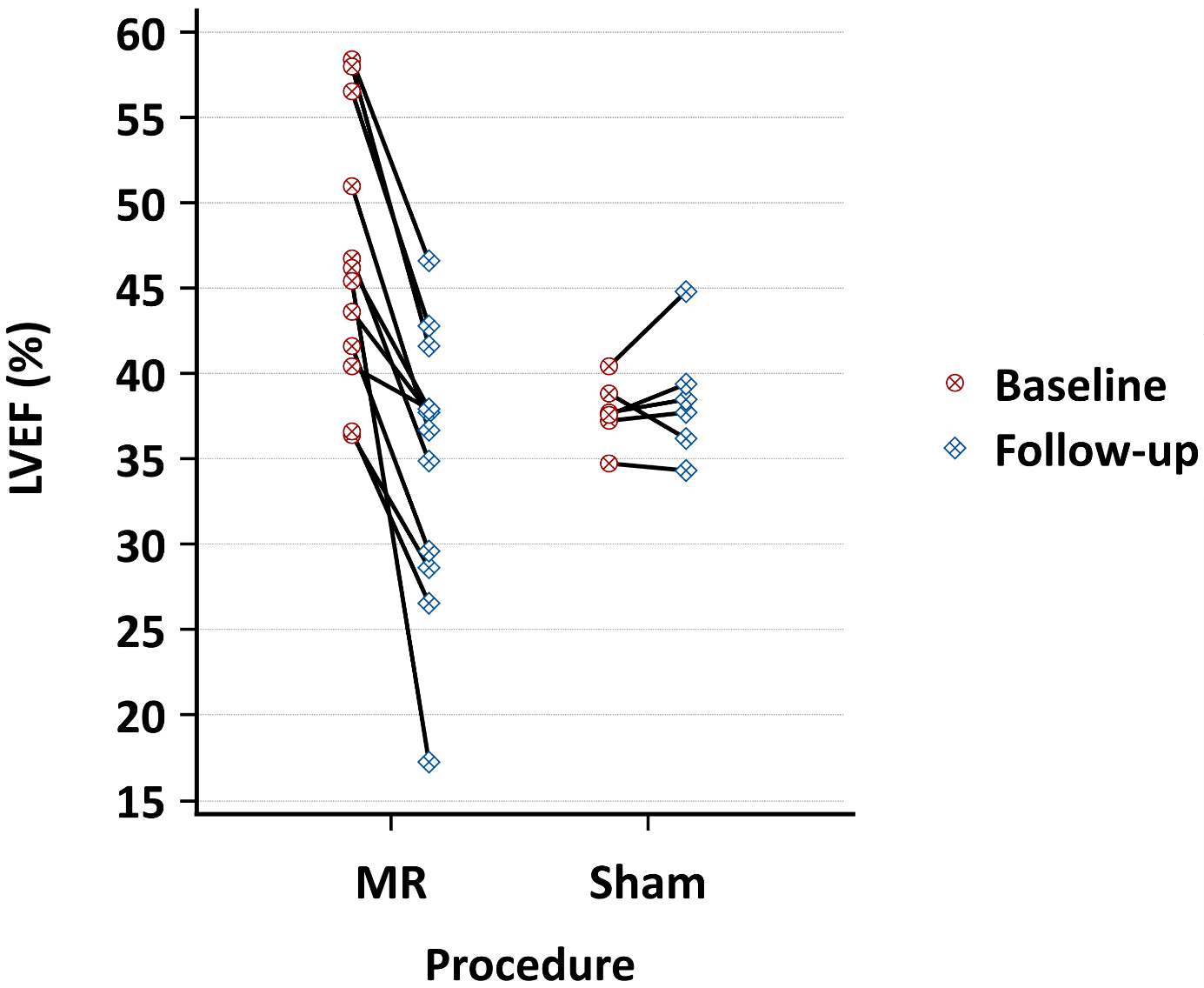


Individual evolution of left ventricular ejection fraction (LVEF) over the four-week follow-up period for the MR group (n = 12) vs. the sham group (n = 6) (p < 0.001; repeated measures ANOVA).

**Supplementary Figure S4**


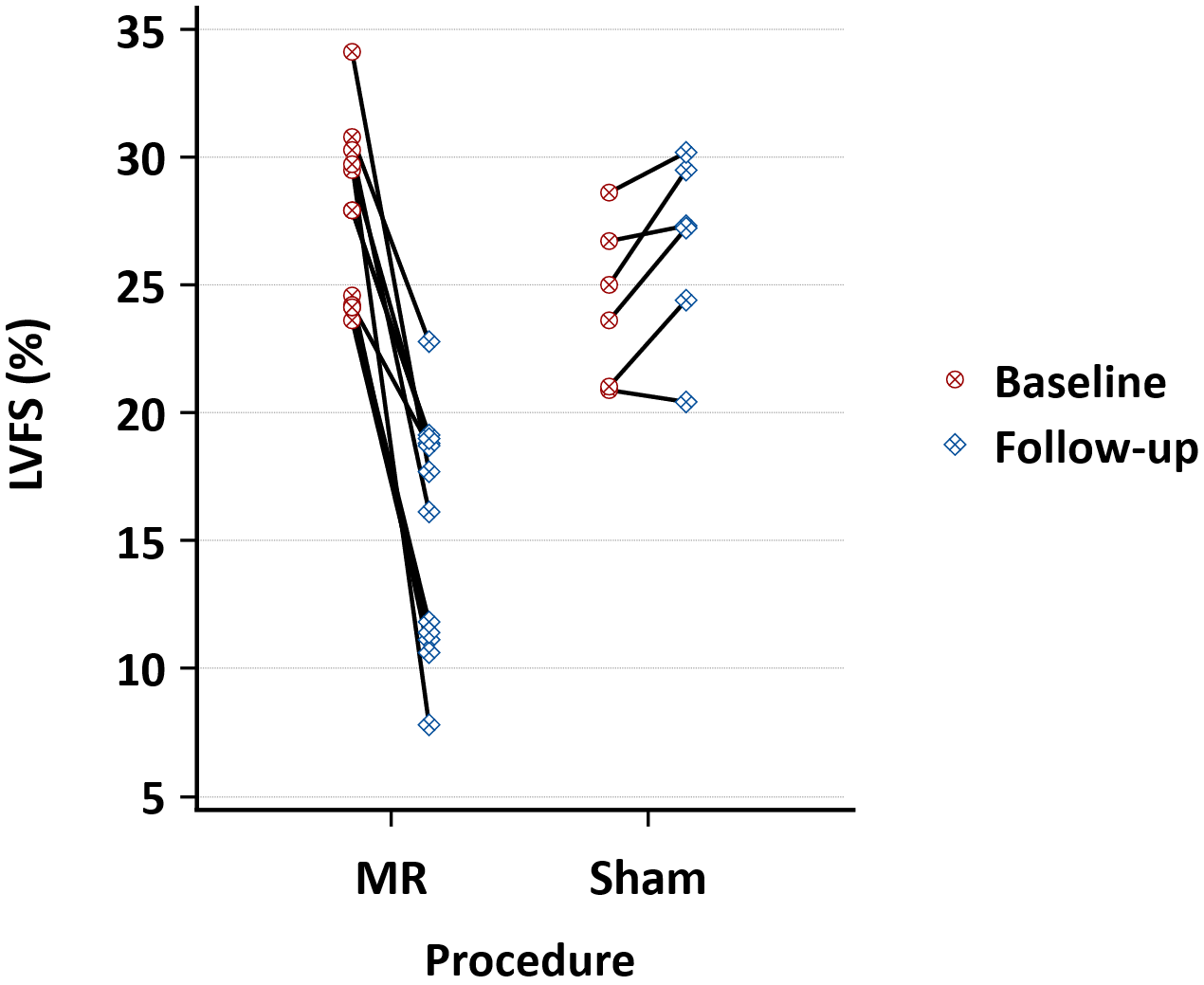


Individual evolution of left ventricular fractional shortening (LVFS) over the four-week follow-up period for the MR group (n = 12) vs. the sham group (n = 6) (p < 0.001; repeated measures ANOVA).
